# Supplementary material for: ProteinShader: illustrative rendering of macromolecules
Source: BMC Struct Biol. 2009 Mar 30;9:19. doi: 10.1186/1472-6807-9-19 (PMC2672931; doi:10.1186/1472-6807-9-19)
Supplement: Additional file 1 — ProteinShader program without source code. This compressed file contains the complete ProteinShader program including associated libraries, but no source code. A README.txt file gives an overview of the ProteinShader distribution, and the index.html file in the help subdirectory has directions on getting started with the program as well as a set of tutorials. [file 1472-6807-9-19-S1.zip › ProteinShader-beta-0_9_4-binary/help/api/org/proteinshader/structure/class-use/Record.html]

Uses of Class org.proteinshader.structure.Record (ProteinShader API)


|  |  |  |
| --- | --- | --- |
| **Overview** \| **Package** \| **Class** \| **Use** \| **Tree** \| **Deprecated** \| **Index** \| **Help** | |  |
| PREV   NEXT | **FRAMES**    **NO FRAMES**     **All Classes** |  |


---


## **Uses of Class org.proteinshader.structure.Record**

| Packages that use Record | |
| --- | --- |
| **org.proteinshader.structure** | Holds the classes that store information from a Protein Data Bank file: Structure, Model, Chain, AminoAcid, Heterogen, Water, Atom, Bond, Helix, BetaStrand, Loop, *etc*. |

Uses of Record in org.proteinshader.structure

| Methods in org.proteinshader.structure that return Record | |
| --- | --- |
| `Record` | `Category.addNewRecord(String recordName)`             Creates a Record, adds it to a hash, and returns the new Record. |
| `Record` | `Category.getRecord(String recordName)`             Retrieves a Record by its name. |
| `Record` | `Description.getRecord(String categoryName, String recordName)`             Retrieves a Record by its Category name and Record name. |

| Methods in org.proteinshader.structure that return types with arguments of type Record | |
| --- | --- |
| `Iterator<Record>` | `Category.iteratorRecords()`             Returns an Iterator for the Records held by this Category object. |

---


|  |  |  |
| --- | --- | --- |
| **Overview** \| **Package** \| **Class** \| **Use** \| **Tree** \| **Deprecated** \| **Index** \| **Help** | |  |
| PREV   NEXT | **FRAMES**    **NO FRAMES**     **All Classes** |  |


---

# *Copyright © 2007-2008*
